# Chicago Medical Society

**Published:** 1884-12

**Authors:** 


### CHICAGO MEDICAL SOCIETY.

The regular semi-monthly meeting of this Society was held in Parlor 44, Grand Pacific Hotel, on Monday evening, September 15, at 8 o'clock. The President, Dr. D. A. K. Steele, in the chair. Dr. L. H. Montgomery, Secretary.

Dr. C. W. Earle read a brief paper on "Congenital Malformation of the Stomach," reciting the case of an infant that lived twelve days. Its life was sustained by very minute quantities of nourishment, he, during the time, being unable to diagnosticate the ailment. And it was only by permission of friends that an autopsy was made, when the trouble was ascertained. It consisted in there being no continuation from the stomach through the pylorus to the intestine, nor was there any communication whatever between the stomach and duodenum. The child's biliary secretion seemed healthy enough; and, as well as the meconium, was passed from the bowels.

The speaker also read a report of a case of bony tumor of the female pelvis and exhibited the specimens of both the above cases. The latter's weight was 3½ pounds. Both of which were believed to be rarely met with. They were accordingly, but informally, discussed.

The Secretary presented the following resolutions and moved their adoption:

WHEREAS, From present reports and indications in foreign countries, cholera and yellow fever (both pestilential diseases) prevail, and as the latter especially is always assuming a threatening attitude toward us, and is not conducive to our national prosperity, nor to public health, and should, if possible, be averted with earnest and efficient sanitary measures, and

WHEREAS, Cholera may make its appearance on this continent ere another twelve months shall elapse, and should, if possible, be averted or restricted to the narrowest limits, therefore,

*Resolved*, That it is the sense of the Chicago Medical Society to have that department of the government relating to public health, recognize the services of able sanitarians who constitute the National Board of Health, for the purpose of coöperating with municipal, State and other organizations of a similar kind, and that a committee of seven (7) members of this Society be appointed by the chair to draft suitable resolutions in behalf of said National Board.

*Resolved*, Furthermore, that this committee present said resolutions to the Congress of the United States, memorializing that body to make a sufficient appropriation for the purpose of said Board for scientific investigation in the prevention and restriction of epidemic, preventable and pestilential diseases.

We believe this action should be promptly taken at the coming session of our national legislature, and that a thorough sanitary organization of the nation should be recognized, and with it absolute enforcement of the best means for the protection of her citizens, and the improvement of our inter-state sanitary condition.

The mover of the resolutions stated that, inasmuch as they had been published on the postal card announcements, every officer and member of the Society, which consisted of about 276 members, had (he presumed) become familiar with the object aimed at in presenting them. He desired a full discussion, and to hear the views of each member present, if time permitted. If they are adopted, broad-minded, philanthropic men in official life will see their value when the first case of cholera appears on our borders, which we trust may never occur, but which is by no means impossible by next June. Then we will wish that a thorough vigilance and a more thorough organization had taken place. The time, therefore, for more effective measures to be taken, is now—months before the probable advent of the disease. It seems to him that the scientific, sanitary and commercial interests of the nation all demand the perpetuation of its National Board of

Health. The resolutions were promptly seconded by Drs. R. E. Starkweather and H. J. Reynolds, and after a lengthy discussion, which was participated in by many members, each of whom favored the object of the resolutions, the motion to adopt unanimously prevailed.

The President stated that he wished a little time to consider the appointment of so important a committee as is called for by the resolutions.

Dr. Edmund Andrews offered the following motion, which was duly seconded by Dr. C. E. Webster: "That this Society hereby express its thanks to Dr. Vincent L. Hurlbut for the gift of a very valuable collection of books and pamphlets, consisting of 300 volumes, to the Library Committee of the Society, for the Chicago Public Library." In behalf of the Library Committee, the speaker continued by stating that there are on the shelves of the city library 1,200 volumes for medical reference. The library authorities have bound, at their own expense, 75 volumes. The report was considered a flattering one, especially when the project to establish a medical department by this Society, to the Public Library, was inaugnrated so recently as last spring. The closing remarks of Dr. Andrews elicited a hearty and unanimous vote of thanks for Dr. Hurlbut.

The chair announced the following gentlemen to constitute the committee as provided for in the resolutions previously adopted: Dr. O. C. DeWolf, Dr. R. E. Starkweather, Dr. L. H. Montgomery, Dr. John Bartlett, Dr. J. H. Etheridge, Dr. A. R. Jackson, Dr. J. H. Hollister.

Dr. H. A. Johnson asked to be excused from serving on the committee, for the reason that he was the representative from Illinois on the National Board of Health, his resignation not having been accepted by the President of the United

States. He took pleasure in extending an invitation to the committee to call upon him at his home, and he would render to its members all the assistance he could, and impart any information he had received from his experience as a member of the National Board of Health. His library, also, was at the disposal of the committee.

Dr. J. H. Etheridge then moved that the committee be authorized to present their resolutions to the Society for a final consideration, and then submit them to Congress.

The motion was carried, and the Society adjourned.

---

Dr. D. A. K. Steele, President. The regular semi-monthly meeting of this Society was held at the Grand Pacific Hotel, on the evening of October 6th, 1884.

Dr. John Bartlett, Chairman of the Committee on National Sanitation, reported that while they had agreed on the substance of the proposed resolutions to be submitted for presentation to Congress, they were not in shape to report to the Society, although signed by most of the members. He therefore asked for further time to consider the matter, which was accorded.

Dr. John H. Rauch, Secretary of the Illinois State Board of Health, had read the resolutions prepared by the committee, and as he had been invited by one of its members to address the meeting on their importance, and that of national coöperation with State and municipal governments in arresting the spread of epidemics, he said that matters should be so arranged that there would be concert of action in all municipal and State Boards with a national health organization; that the national government should have control of inter-state quarantine, he had no doubts. The trouble all arises out of inefficient maritime quarantine. Illinois was specially interested in this, from the St. Lawrence river to the Rio Grande river, and from the Atlantic ocean to the Pacific ocean, for this State pays more internal revenue tax than any other State in the Union, New York not excepted. Cholera may arrive at Montreal or Quebec, and be brought to this city and State over the Grand Trunk railroad, the Michigan Central railroad, or Michigan Southern railroad, and we have authority to stop these trains at the State line only, and prevent them entering the State of Illinois. New York controlled that port exclusively, and Illinois had no authority to interfere, while her interest in keeping out diseases was just as great as that of New York. The Illinois Board of Health was prepared to prevent the entry of infectious and contagious diseases, no matter what contingency might arise, but it would be better if she could depend upon the aid of her sister States who were equally interested. Two-thirds of the number of emigrants coming to this country arrive at the port of New York. The emigrant inspection service carried on by the National Board of Health a few years ago, under the auspices of the national government, was done at a cost of only $50,000, when some forty odd thousand emigrants were vaccinated on the trains, and there was no detention. The National Board of Health no longer exists except in name, the last Congress having cut off all appropriations, consequently we are not in as good shape to ward off epidemics as we were three or four years ago. An endeavor to protect the States cannot be well done, on account of inefficient maritime quarantine, and the only resource we have at present to cope with these diseases is for the different States to act in good faith and compact with each other. An illustration was cited by the speaker to prove his statement correct, where, in 1878-79, along the banks of the Mississippi and Ohio rivers, epidemics prevailed; that since the organization of the Sanitary Council of States along these rivers, infectious diseases and all other diseases are much less.

The speaker then recited the history of the National Board of Health, its origin, the inefficiency of our laws, etc., and cited as further illustration how inadequate the surgeon of this port and the one at Cairo would be to antagonize or cope with any form of epidemic ; that instead of this being only under the control of the State, it should be controlled by the national government. Now is the time to prepare to secure legislation, instead of waiting until after the heels of an epidemic, as has been done heretofore. Those who have charge of sanitary interests in the different States should study the causes of disease, investigate the subject, and through thorough coöperation with a national health bureau we would be well prepared to prevent the advent of cholera next year. The machinery of no one man can be made to work or control the state of commerce satisfactorily, nor can this be done by one State alone ; more States should be represented, and concert of action of all those in authority should prevail. The United States Treasury is entirely independent of States in this respect. A conference of the boards of health of the several States and Territories will be held at St. Louis next week, having for its object the coöperation of all authorities for the purpose of controlling epidemics and contagious diseases. He thought that some plan would be evolved that would prove more satisfactory to all, and do away with the petty jealousies that have hampered the action of the national health bureau. The general feeling prevails that we are going to have cholera in this country next year, and now is the time to prepare for it as the resolutions call for. Dr. J. H. Hollister said that as a member of the committee he should decline to attempt to instruct Congress as to its duties in the matter, but thought that a memorial presented by this Society would be the better mode of expression. If our memorial to Congress is only to express a desire as to what is most wanted by the public, then we, as medical gentlemen, have done our part. It is a subject that takes hold of common interests, and how to render efficient aid suitably but not unnecessarily is something that requires more wisdom than can be gained by temporary discussion. He thought our wisest men should meet in conference and unite on some practical method and then urge legislative action. The speaker asked Dr. Rauch: to what extent are States moving in this direction of having State Boards of Health organized? and to what extent does their authority reach? He was answered, that Massachusetts, New Hampshire, Connecticut, New York, New Jersey, Maryland, West Virginia, Virginia, Indiana, Illinois, Michigan, Kentucky, Tennessee, Louisiana, Mississippi, Arkansas, Alabama, Iowa, Wisconsin, Minnesota and Missouri, each have their State Board of Health. Ohio, Pennsylvania, Maine and Vermont have no State Boards of Health. Lower Canada has none. But that the Ontario Board of Health is in good shape. The Michigan Board of Health is an advisory one. The Illinois Board of Health controls quarantine and can call on sheriffs and constables to obey her dictation. The boards of Missouri and Minnesota are nearly like ours, New York's board is partly like ours in authority. Indiana's board is nearly like ours, only better, for they have County Boards of Health. The Boards of Health of Maryland and Virginia are also very efficient. Dr. L. H. Motgomery alluded to the merits of the National Board of Health, to the efficient services it had rendered in the past, and hoped it would be adequately provided for in the future. He had spoken to the Representative in the Congressional district where he resides upon the subject, and was informed by the member that he would be glad to do his part and use his influence toward securing the granting of legislation for the establishment of some form of a national sanitary organization. The speaker was also assured by the honorable gentleman that he had been, and at present is, a friend of the National Board of Health, for its services were performed with a degree of celerity and exactness that pleased everybody in the States. Regarding memorializing Congress, as Dr. Hollister alluded to, there is a clause in the resolution providing for this.

Every State and Territory in the Union should be represented on this board by the appointment of some one representative medical gentleman to it, for, as Dr. DeWolf said, inter-state observation. Indeed, we might do more, if needs be, than transmit a memorial to Congress; but petition, or appeal to our national legislators to appropriate sufficient means and appliances to sustain some form of National Health Association, which he believed is the sentiment of at least nine-tenths of the physicians and all the people.

Dr. R. E. Starkweather had hoped that the committee were prepared to submit their resolutions this evening. He, as one of its members, endorsed every word they contained, even at the risk of their being (as possibly they were in one place) tautological. He hoped the committee would be able to perfect them before the close of the week. Dr. Rauch, in conclusion, stated that he intended to speak to some extent upon this subject at the meeting of the State Boards of Health to be held at St. Louis next week. A special meeting will probably be held at Washington in December of all the State sanitary boards, at which time this subject will receive special attention. The Society tendered a vote of thanks to Dr. Rauch for his address and information given. The committee on resolutions was requested to meet immediately after the close of this meeting, which was then adjourned.

